# Flexibility in Language Action Interaction: The Influence of Movement Type

**DOI:** 10.3389/fnhum.2018.00252

**Published:** 2018-06-25

**Authors:** Zubaida Shebani, Friedemann Pulvermüller

**Affiliations:** ^1^Cognition and Brain Sciences Unit, University of Cambridge, Cambridge, United Kingdom; ^2^Linguistics Department, College of Humanities and Social Sciences, United Arab Emirates University, Al-Ain, United Arab Emirates; ^3^Brain Language Laboratory, Department of Philosophy and Humanities, Freie Universität Berlin, Berlin, Germany; ^4^Berlin School of Mind and Brain, Humboldt Universität zu Berlin, Berlin, Germany; ^5^Einstein Center for Neurosciences, Berlin, Germany

**Keywords:** action word, working memory, motor movement, motor-language interaction, semantics

## Abstract

Recent neuropsychological studies in neurological patients and healthy subjects suggest a close functional relationship between the brain systems for language and action. Facilitation and inhibition effects of motor system activity on language processing have been demonstrated as well as causal effects in the reverse direction, from language processes on motor excitability or performance. However, as the documented effects between motor and language systems were sometimes facilitatory and sometimes inhibitory, the “sign” of these effects still remains to be explained. In a previous study, we reported a word-category-specific differential impairment of verbal working memory for concordant arm- and leg-related action words brought about by complex sequential movements of the hands and feet. In this article, we seek to determine whether the sign of the functional interaction between language and action systems of the human brain can be changed in a predictable manner by changing movement type. We here report that the sign of the effect of motor movement on action word memory can be reversed from interference to facilitation if, instead of complex movement sequences, simple repetitive movements are performed. Specifically, when engaged in finger tapping, subjects were able to remember relatively more arm-related action words (as compared to control conditions), thus documenting an enhancement of working memory brought about by simple hand movements. In contrast, when performing complex sequences of finger movements, an effector-specific degradation of action word memory was found. By manipulating the sign of the effect in accord with theory-driven predictions, these findings provide support for shared neural bases for motor movement and verbal working memory for action-related words and strengthen the argument that motor systems play a causal and functionally relevant role in language processing semantically related to action.

## Introduction

The relationship between language and action has sparked much interest in recent years and findings suggest a close connection between language and motor function. Evidence derived from a range of different methodological approaches including neuroimaging, neurophysiological and behavioral investigations demonstrates the involvement of motor systems in the processing of action-related language (Fadiga et al., 2002; Glenberg and Kaschak, 2002; Hauk et al., 2004; Shtyrov et al., 2004, 2014; Buccino et al., 2005; Pulvermüller et al., 2005; Boulenger et al., 2006, 2009; Fischer and Zwaan, 2008; Glenberg et al., 2008a; Dalla Volta et al., 2009; D’Ausilio et al., 2009; Schuil et al., 2013; Trumpp et al., 2013; Klepp et al., 2014; Gianelli and Dalla Volta, 2015; Grisoni et al., 2016; Vukovik et al., 2017; see Króliczak et al., 2011; Vingerhoets et al., 2013; Crivelli et al., 2018). Neuroimaging studies have found motor systems to be active during the processing of single action words (e.g., Hauk et al., 2004), action-related concrete sentences (Tettamanti et al., 2005; Aziz-Zadeh et al., 2006) and even action-related idiomatic language (Boulenger et al., 2009, 2012), but see Desai et al. (2013) for different results. Interestingly, several, although not all studies (for reviews see Kiefer and Pulvermüller, 2012; Meteyard et al., 2012; Glenberg et al., 2013; Dijkstra and Post, 2015; Kemmerer, 2015a), found that motor system activations reflected the *meaning* of action-related language. For example, words that refer to actions typically performed by the face, arm and leg have been found to somatotopically activate the very same areas in the motor and premotor cortex that control movement of those specific body parts, with face-related action words, such as *chew*, activating inferior-frontocentral areas that control articulation and face movements, arm-related action words, such as *grasp*, activating the cortical area that controls hand/arm movements and leg-related action words, such as *step*, activating the dorsal cortical area that controls foot/leg movements (Hauk et al., 2004, 2008; Shtyrov et al., 2004, 2014; Kemmerer and Gonzalez-Castillo, 2010; Willems and Casasanto, 2011; Kemmerer, 2015a; Grisoni et al., 2016). However, not all studies addressing semantic functions agree on a semantic contribution of cortical motor systems, in part because of separate evidence that the same areas contribute to phonological and possibly morphological processes (Pulvermüller et al., 2006; de Zubicaray et al., 2013; Schomers et al., 2015; Schomers and Pulvermüller, 2016), and in part because experiments trying to replicate previously documented effects did show somewhat different results (Postle et al., 2008, 2013; Kemmerer, 2015b).

Some findings from neuropsychology have been interpreted as support for the claim that the brain systems for language and action are tightly interconnected. If action language processing relies on motor systems, then cortical lesions in motor regions should affect the processing of action-related language. Indeed, nouns and verbs have been found to be differentially affected in patients with lesions due to stroke or neurodegenerative disease (Damasio and Tranel, 1993; Bak et al., 2001; Neininger and Pulvermüller, 2003; Cotelli et al., 2006; Boulenger et al., 2008; Kemmerer et al., 2012; Dreyer et al., 2015). For example, patients with motor neuron disease, a neurodegenerative condition characterized by atrophy in primary motor and premotor cortex, were found to be more impaired on action verbs than object nouns (Bak et al., 2001; Bak and Chandran, 2012). Similar results were found in patients with the frontal variant of frontotemporal dementia (Cotelli et al., 2006) and Parkinson’s disease, a condition characterized by motor disorders (Boulenger et al., 2008). In sum, selective action verb or tool noun deficits following focal lesions in motor regions have been interpreted as strong evidence for the importance of motor systems in the semantic processing of action related language. Alternative positions try to tone down the causal effect of motor systems on semantic processing and emphasize instead the role of central “semantic hubs” (for alternative views, see Mahon and Hickok, 2016). However, the possible existence of one or more “semantic hubs” is independent from a possible complementary role of sensory and motor systems in semantic processing (Pulvermüller et al., 2014; Shebani et al., 2017).

Findings from neuroimaging and neuropsychology documenting the functional interaction of language and motor systems of the brain are further substantiated by investigations of transcranial magnetic stimulation (TMS) and action execution performance. Some of these behavioral neuropsychological studies demonstrate facilitation effects of motor systems activity on language processing, whereas others show inhibitory effects (see for example, Glenberg and Kaschak, 2002; Pulvermüller et al., 2005; Glenberg et al., 2008a; D’Ausilio et al., 2009; Willems et al., 2011; Repetto et al., 2013; Vukovik et al., 2017). For instance, shorter reaction times to body-part specific subtypes of action words have been found when TMS is applied to hand and leg motor cortex (Pulvermüller et al., 2005) and after theta burst TMS is applied to the hand area of the premotor cortex (Willems et al., 2011). Showing inhibition effects, Glenberg et al. (2008a) report that moving objects either towards or away from the body slowed the processing of “towards” or “away” sentences. Kaschak et al. (2005) found that even the perception of motion in a particular direction selectively impaired processing of sentences that describe motion in that same direction. Facilitation and inhibition effects have also been demonstrated in the reverse direction, from language processes on motor excitability or performance (Fadiga et al., 2002; Buccino et al., 2005; Scorolli and Borghi, 2007; Glenberg et al., 2008b; Sato et al., 2008; Dalla Volta et al., 2009; Liepelt et al., 2012; Gianelli and Dalla Volta, 2015). For instance, Sato et al. (2008) report slower reaction times to arm-related action words when subjects used a hand movement to indicate a response. Likewise, in a semantic task, Dalla Volta et al. (2009) found that arm- and leg-related action words interfered with an action when it was executed using the same effector. In testing whether action language interactions are bidirectional, Liepelt et al. (2012), found an effect of action word processing on executing a hand movement and the reverse, an effect of perceived hand movement on action word production.

However, some of the investigations of motor-language interaction report inconsistent results with regards to the sign of the effect, that is, whether the causal influence is positive and facilitatory, or negative and inhibitory. For example, the study by Buccino et al. (2005) showed that listening to action related language results in longer reaction times and reduces the amplitude of TMS-induced motor evoked potentials (MEPs) recorded from hand and leg muscles when TMS is applied (see also Fadiga et al., 2002). Other articles have confirmed inhibition effects on hand related action words processing when repetitive TMS was applied (see for example, Repetto et al., 2013; Vukovik et al., 2017). In contrast to these inhibitory results, other studies reported facilitation effects and relatively shorter reaction times to language materials with TMS stimulation to motor systems (for example, Pulvermüller et al., 2005; Glenberg et al., 2008b; Willems et al., 2011) including a study by Gianelli and Dalla Volta (2015), the aim of which was to replicate the *inhibition* effects reported in Buccino et al. (2005). Replication failures and unpredictable “signs” of the effects were also reported in the study of words (usually nouns) whose referent objects afford actions (e.g., a “cup”, which affords grasping and drinking (Zwaan and Pecher, 2012; Pecher, 2013). Even though the majority of studies on language-action interaction report positive results when genuine action words (verbs) and sentences are probed, the factors influencing the direction of the effect (facilitation or interference) are still not well understood. More definitive tests are required to assess whether features such as TMS stimulation strength and/or length, the nature of motor movement and/or the similarity between word-related action scheme and to-be-performed motor activity, co-determine the sign of the interaction effect.

Critics of semantic grounding approaches postulating an intrinsic functional link between brain systems for language and actions have taken these different effects as indication for inconsistency or lack of replicability, thus suggesting that, overall, no clear and replicable effects emerged from the literature. However, this type of criticism seems to be based on a simplistic assumption, namely that neuronal connections must exert the same influence independent of the system’s states and previous activations. We argue here that sophisticated neurobiological models may well explain aspects of the observed “flexibility” of action language interactions. In doing so, we will focus on the sign of causal motor-language effects.

In examining the effects of action word processing on overt motor behavior, some studies managed to successfully influence the sign of the effect by manipulating the timing of action language interaction (e.g., Boulenger et al., 2006; de Vega et al., 2013). Using continuous analyses of fine grained movement kinematics while participants were engaged in a language task, Boulenger et al found that processing action words hindered the execution of a reaching movement when performed concurrently, while it assisted subsequent motor performance when performed prior to the onset of the reaching movement. Their results demonstrate that action word processing can facilitate or interfere with motor behavior depending on the temporal relationship between motor and linguistic processes. Consistent with this, de Vega et al. (2013) showed that the processing of towards/away transfer sentences either primed or interfered with a towards/away motor movement depending on the degree of temporal overlapping between language and action. Shorter intervals between the onset of the transfer verb and the cue for motor action (stimulus onset asynchrony, SOAs: 100 and 200 ms) interfered with toward/away movement while a longer SOA (350 ms) positively primed the directional motor response. These studies concluded that when action language processing occurred simultaneously with motor movement (Boulenger et al., 2006) or temporally close to motor movement (de Vega et al., 2013), the observed interference effects arose from competition between the two motor programs for shared processing resources. This is consistent with the statement that aspects of the meaning of action related words are motor schemes cortically processed and represented, at least in part, in the motor system of the brain, including motor and premotor cortex (Pulvermüller, 2001, 2005). In this neurosemantic framework, two incompatible motor schemes would inhibit each other by way of local inhibitory connections in cortex (for a formal model, see Garagnani et al., 2008; Tomasello et al., 2017). Therefore, processing action words and performing a movement using the same effector as would be used in the execution of the actions related to the words being processed would lead to interference effects. The facilitation effects observed in Boulenger et al. (2006) and de Vega et al. (2013), on the other hand, can be explained in terms of action word processing pre-activating the motor network, thereby priming the execution of the motor movement. Because the facilitation effects were found when the processing of action words directly preceded the motor movement, lingering activation in the motor network may have facilitated the execution of the subsequent reaching movement.

Boulenger et al. (2006) and de Vega et al. (2013) have shown that the sign of the effect can be successfully manipulated in examining the temporal relationship between language processes and motor performance. Kaschak et al. (2005) suggest that integratibility, or the extent to which the content of the sentence and the perception of movement are integratible, is also a factor in determining the sign of the effect. We investigate here a third possibility, that the sign depends on the complexity of motor movements that are processed together with linguistic and semantic information. In Shebani and Pulvermüller (2013), we describe an *impairment* of working memory for arm- and leg-related action words as a result of complex sequential movements of the hands and feet. Subjects were more impaired on remembering arm-related action words when performing a complex rhythmic motor sequence with their hands, while they were more impaired on recalling leg-related action words when performing the complex, demanding movements with their feet. The results demonstrate that sensorimotor brain systems can exert an inhibitory effect on action-word memory, suggesting that motor systems play a causal and necessary role in action-language processing. The finding also suggests that different subparts of the motor system are susceptible to functional changes caused by complex sequential motor movements. Two complex motor movements are obviously incompatible with each other: I cannot perform a drumming exercise while at the same time grasping a pen. It is therefore well motivated to postulate inhibition effects between such complex motor tasks and also between performance of one complex motor schema and activation of a different complex motor schema semantically related to an action word (see Shebani and Pulvermüller, 2013). However, a simple motor movement such as contracting a finger muscle may be *part of* many more complex movement sequences: Tapping my finger may therefore not compete with grasping but, instead, pre-activate part of the “grasping” circuit. Following this line of thought, one can predict facilitation effects between simple and complex motor schemas, and, thus, likewise, facilitation effects of elementary finger movements on the processing of action words semantically related to complex actions.

To test whether the pattern of interaction observed in Shebani and Pulvermüller (2013) can be reversed, the following experiment was designed. Using the same working memory paradigm as outlined in Shebani and Pulvermüller (2013), we examined the ability of subjects to remember lists of arm- and leg-related action words while performing a simple, continuous motor sequence with their hands and feet. The prediction was that memory for concordant arm- and leg-related action words would significantly improve with motor movement.

## Materials and Methods

### Participants

Twenty-six native speakers of English (14 females) aged 18–30 (mean = 21.8, SD = 3.5) took part in the experiment. All reported normal hearing and normal or corrected-to-normal vision and had no history of neurological or psychiatric illness. All participants were also right-handed with an average laterality quotient of 78% (SD = 18.8), from a reduced version of the Oldfield handedness inventory (Oldfield, 1971). Participants were screened for drumming experience or any other activity that requires excessive amounts of motor independence and coordination (drummers were excluded from the study). The sample size was determined based on a standard of comparable studies (e.g., Sato et al., 2008; Gianelli and Dalla Volta, 2015; Vukovik et al., 2017). All subjects gave written informed consent prior to their participation and were reimbursed for their time. Ethics approval was obtained from the Cambridge Local Research Ethics Committee.

### Material

The lexical stimuli used in the present experiment were similar to those used in Shebani and Pulvermüller (2013) and consisted of 48 words, 24 arm-related action words and 24 leg-related action. As in Shebani and Pulvermüller (2013), lexical stimuli were closely matched for a range of psycholinguistic and semantic variables including number of letters, number of phonemes, standardized lexical frequency, lemma frequency, letter bigram frequency, letter trigram frequency, grammatical ambiguity, valence, imageability and general action relatedness (see Table 1). The two word groups differed significantly only on semantic arm- (5.77 vs. 1.88) and leg-relatedness (2.29 vs. 5.89). Two pseudo-randomized stimulus sequences were used in the experiment, alternated between subjects.

**Table 1:** Means and standard deviations of psycholinguistic properties for arm and leg words.

Psycholinguistic feature	Arm words		Leg words	
	Mean	SE	Mean	SE
Number of phonemes	3.63	(0.13)	3.92	(0.17)
Number of letters	4.38	(0.16)	4.70	(0.15)
Grammatical ambiguity	1.96	(0.04)	1.96	(0.04)
Word frequency	247.7	(70.9)	242.5	(71.2)
Lemma frequency	584.4	(121.3)	583.5	(143)
Bigram frequency	29013	(3331)	29446	(3370)
Trigram frequency	2995	(427.8)	2650	(360.8)
*Valence	3.73	(0.19)	4.22	(0.17)
Arousal	3.10	(0.19)	3.37	(0.17)
Imageability	4.79	(0.15)	4.79	(0.14)
Visual relatedness	4.60	(0.17)	4.39	(0.16)
Body relatedness	3.94	(0.19)	4.15	(0.13)
Action relatedness	5.33	(0.18)	5.50	(0.16)

Differences between arm and leg words were n.s. at p < 0.05. *The difference in valence between arm- and leg-related words was close to significance, but did not reach significance criteria.

### Procedure

The procedure was identical to that of Shebani and Pulvermüller (2013) apart from the motor sequence in the hand movement and foot movement conditions being simpler and substantially less challenging. Again, there were four conditions in the experiment (control, hand movement, foot movement and articulatory). In each condition, a fixation point was presented alone in the center of the screen for 3 s, after which it was replaced with four words presented serially. The words presented in each trial were either all arm-related or all leg-related action words. Each word was presented for 100 ms. The SOA of two subsequent stimuli was 500 ms (two words per second). Stimulus presentation and encoding was followed by a 6 s memory period during which subjects were required to keep the four words in memory in the order in which they were presented. After this delay, a beep prompted subjects to repeat the words they saw on screen.

Subjects received instructions for each of the four conditions. In the control condition, subjects were asked to wait silently while keeping the words in memory during the 6 s delay until they heard the beep prompting them to repeat the words previously presented on screen. In the hand movement condition, in addition to retaining the words in memory, subjects were required to alternate tapping their index fingers on the table before them rapidly and continuously during the 6 s memory period. Subjects were instructed to start tapping as soon as the fourth word disappeared at the end of the encoding period and continue tapping until they heard the beep prompting word retrieval. Similarly, in the foot tapping condition, subjects were required to tap their feet rapidly and continuously while keeping the words in memory during the delay period. In the articulatory condition, subjects were instructed to repeat the syllable [ba] continuously and at an even pace (approximately two words per second) while keeping the words in memory during the delay period. The purpose of the articulatory condition was to occupy auditory/verbal working memory systems and so prevent verbal recoding/rehearsal of the visually presented words (Baddeley, 1986). The experimenter was present during all testing sessions to ensure that the tasks of the articulatory, hand movement and foot movement conditions were being carried out as instructed.

The four conditions were run as separate blocks with 24 trials in each block, 12 arm-related word trials and 12 leg-related word trials. Trial presentation was self-paced; subjects initiated each trial by pressing the space bar of a computer keyboard before them. Stimulus items were presented in a different random order in each trial. The full set of 48 words was presented twice in each of the four conditions. Arm- and leg-related word trials were randomized within each block with the constraint that not more than three trials of the same word category appeared consecutively. The order of the blocks was counterbalanced across subjects using a Latin-square design. Clear written and verbal instructions were given to all subjects before starting the experiment and again just prior to each block. Enough opportunity for practice was given so that subjects were acquainted with the encoding, memory and retrieval stages of the experiment. Subjects did not begin each block until they and the experimenter were satisfied that they understood the instructions and performed the simple motor movements as instructed. Breaks were encouraged between blocks and within blocks if needed.

### Statistical Analysis

Number of errors made in the four conditions (control, hand movement, foot movement and articulatory) and two word categories (arm- vs. leg-related words) were obtained for each participant and submitted to a 2-way repeated measures Analysis of Variance, ANOVA (Word Type × Condition) for statistical analysis. Further ANOVAs were carried out on subsets of conditions and further F-tests were done for Planned Comparison testing. Analyses of the different error types (omission, replacement and transposition/shift) were also performed.

## Results

**Figure 1:**
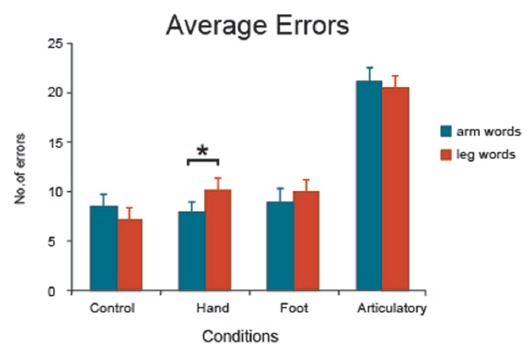
Average errors made on arm- and leg-related words and standard error measures in each experimental condition (control, hand movement, foot movement and articulatory). Finger tapping led to enhanced memory performance for arm-related words in comparison to leg-related words. *Indicates significance.

The different error types were calculated for each subject and averaged separately. The majority of errors were replacements (52%) and omissions (29%); transposition/shift errors were less frequent (19%). The Analysis of Variance on overall errors revealed a significant main effect of condition (*F*_(3,75)_ = 97.7, *p* < 0.0001) with most errors made in conditions with motor movement, and a significant interaction effect (*F*_(3,75)_ = 2.93, *p* < 0.04). Planned comparison analyses revealed no significant word category differences in the control (*F*_(1,25)_ = 2.79, *p* = 0.11) and articulatory conditions (*F* < 1). However, when subjects engaged in simple finger tapping, a significant word category difference emerged (*F*_(1,25)_ = 8.25, *p* = 0.008), with less errors made on arm-related than on leg-related action words (7.9 arm word vs. 10.2 leg word errors; Figure 1). The analysis failed to reveal a between-category difference in the foot movement condition (*F* < 1) with almost the same number of errors made in the two word categories (9.0 arm vs. 9.9 leg word errors, difference n.s.). Crucially, a significant interaction effect was found in the comparison of data from the hand movement and control conditions (*F*_(1,25)_ = 9.24, *p* < 0.006) and even after removal of shift and transposition errors from the analysis, this critical interaction effect remained highly significant (*F*_(1,25)_ = 8.01, *p* < 0.009).

When data was normalized to reduce variance between subjects, the z-transformed data confirmed all significant interactions and statistical differences. Normalized data revealed a significant Word Type × Condition interaction (*F*_(3,75)_ = 2.77, *p* < 0.047) and a significant interaction in comparing the hand movement and control condition (*F*_(1,25)_ = 10.83, *p* < 0.003). The critical word category difference in the hand movement condition remained highly significant (relatively less errors on arm-related words *F*_(1,25)_ = 8.49, *p* = 0.007), while there were no significant word category differences in the other three experimental conditions.

A large selection of subjects was tested in the experiment (*n* = 26) including bilingual subjects and those who reported left-handed family members. A number of subjects tested also made very few errors (e.g., 15%). To obtain a more homogenous sample of subjects and data and to parallel selection criteria between Shebani and Pulvermüller (2013) and the present experiment, an additional analysis was performed. Bilingual subjects (*n* = 2) and subjects with left-handed family members (*n* = 6) were removed from this analysis along with subjects whose error scores did not reach a cut-off threshold set to a minimum of 15 errors in at least one category of one condition (*n* = 4). When data from these subjects (*n* = 12) were excluded from the analysis, all critical interactions and planned comparisons remained the same. Thus, all statistical differences were confirmed even after removal of these atypical and high performing subjects.

## Inter-Experimental Analyses

**Figure 2:**
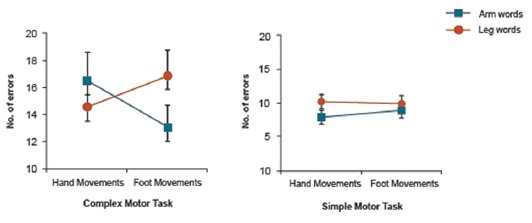
Three-way interaction of Word Category × Motor Movement × Task Complexity interaction in the experiment reported in Shebani and Pulvermüller (2013; complex motor task) and the present study (simple motor task). When simple movements are carried out, the inhibitory effect of motor activity on body-part-congruent language memory is reversed for arm-related words.

Results of the present study were compared with those reported in our earlier study (Shebani and Pulvermüller, 2013). As different sample sizes were used in the two experiments (*n* = 15, *n* = 26), Levene’s test of equality of variances was used to test whether homogeneity of variances applied across these studies. Results indicate that variances were not significantly different in the two groups. This was true for performance in the control condition on both arm-related words (*F* = 1.07, *p* = 0.31) and leg-related words (*F* = 0.92, *p* = 0.34). Results of the two experiments were entered into a 2 × 2 × 2 ANOVA with the factors Word Category (arm/leg), Motor Movement (hand/foot) and Task Complexity (complex/simple). The analysis revealed a significant main effect of Task Complexity (*F*_(1,39)_ = 10.75, *p* = 0.002) and a significant three-way interaction effect (*F*_(1,39)_ = 9.6, *p* < 0.004, Figure 2).

**Figure 3:**
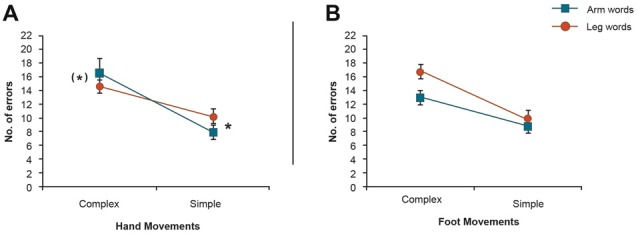
2 × 2 Analysis of variance (ANOVAs) of the movement conditions in both experiments. **(A)** Cross-over Word Category × Task Complexity interaction in the hand movement conditions showing a significant Word Category difference in the complex motor task. This result demonstrates that changes to the complexity of the motor task reversed the pattern of interaction from interference to facilitation in the hand movement condition. **(B)** Word Category × Task Complexity interaction in the foot movement conditions. *Indicates significance. (*)Indicates near significance.

At first glance, this interaction seemed to be due to the fact that the inhibitory, damaging effect of motor activity on body-part-congruent language memory is reversed, in the case of arm-related words, when simple movements are being carried out. However, as this complex effect could be due to different aspects of the data, we unpacked the 3-way interaction. We first focused on the hand movement conditions of both experiments (design: Word Category (2) × Task Complexity (simple/complex)). Apart from a significant main effect of Task Complexity (*F*_(1,39)_ = 10.57, *p* = 0.002), this analysis showed a significant cross-over Word Category × Task Complexity interaction (*F*_(1,39)_ = 10.68, *p* = 0.002, Figure 3A). Furthermore, paired *t*-tests in the hand movement conditions revealed a significant word category difference in the simple motor task (*F*_(1,39)_ = 8.65, *p* < 0.006) and a near significant difference in the complex motor task (*F*_(1,39)_ = 3.50, *p* < 0.069). A similar analysis was also performed for the foot movement conditions of both experiments, which yielded significant main effect of Task Complexity (*F*_(1,39)_ = 7.57, *p* < 0.009) and Word Category (*F*_(1,39)_ = 6.60, *p* < 0.014), but no strong support for a Word Category × Task Complexity interaction effect (*F*_(1,39)_ = 2.34, *p* < 0.13, Figure 3B).

**Figure 4:**
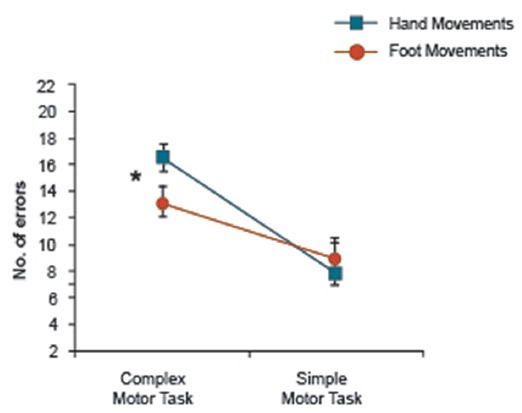
Motor Movement × Task Complexity interaction of arm-related words only in both experiments showing a significant Motor Movement difference in the complex motor task. *Indicates significance.

A further inter-experimental analysis was conducted on data from both hand and foot movement conditions of the two experiments for arm-related words only (Movement Type (2) × Task Complexity (2)). The 2 × 2 ANOVA revealed a significant interaction of Motor Movement (hand/foot) × Task Complexity (*F*_(1,39)_ = 5.60, *p* < 0.023), with planned comparisons revealing more errors in the complex task during hand movements compared with foot movements (Figure 4). In summary, complex hand movements impaired memory for arm-related action words, but not for leg-related action words and simple hand movements tended to assist memory for arm-related action words, but simple foot movements did not enhance leg word memory.

## Discussion

The aim of the present study was to scrutinize the factors determining the flexibility of the causal effects exerted by the motor system on the language system. In particular, the “sign” (facilitatory vs. inhibitory) of the influence of motor movement on the processing of action-related verbs was studied in a verbal working memory task. More specifically, we tried to determine whether the choice of the distractor motor movements executed during action word memory can alter the sign of the effect, so that, in contrast to previously reported inhibitory influence of complex motor activities on action word verbal memory, elementary and repetitive movement leads to facilitation of verbal working memory for action related words.

Our results did not show a full inversion for repetitive simple hand/leg movements on hand and leg-related action verbs. However, the predicted inversion effect could be confirmed for simple hand movements, which improved memory performance for arm action words, as documented by significant interactions of the Motor Movement and Task Complexity factors, as well as the Word Category and Task Complexity variables. As illustrated in Figure 1, the results demonstrate that when subjects engaged in simple repetitive and highly automatized finger alterations during action word memory, they were able to remember relatively more arm-related words than leg-related words. This result is further strengthened by the significant Word by Movement type interaction emerging from the comparison of arm-word and control conditions. Fewer errors on arm-related words than on leg-related words in the hand movement condition is open to the interpretation that finger tapping facilitated memory for arm-related action words relative to leg words. The reversal of the effects (facilitation vs. inhibition) of simple and complex motor performance on the processing of arm related verbs was documented by further statistically significant interaction effects, which emerged when comparing the results of two different experiments using the same word stimuli but different tasks, a complex paradiddle drumming task and the simple finger alternation task.

Why did our experiments fail to show similar facilitation and reversal effects of foot movements on leg word memory as they emerged for arm-related action words and finger movements? Among the many possibilities that could underlie this null result, we would like to mention just two: The alternating foot movement condition applied in our study required subjects to perform a sequence of leg movements which are similarly performed during walking. Now, walking is a highly automatized activity which may therefore require very little cortical activity, thus decreasing the likelihood of measurable effects. Furthermore, we are used to talking while walking so this practice related skill may work against finding effects in this condition. However, we would like to remind the reader that, in our previous study, we found interference effects of complex drumming performance involving unfamiliar movement using the feet and the processing of leg-related action words. Therefore, the lack of differential effects in the simple foot movement condition may be a result of foot tapping not producing enough activation or of previous learning to process language while walking.

Some critics of grounded cognition and “embodied semantics” have argued against the “flexibility” of any causal influences between sensorimotor and language processes. In essence, the argument seems to be that, if not all experiments show the same facilitation or inhibition, which occurs across tasks, conditions, stimuli and experiments, any results must be uninterpretable or at least at variance with theories postulating a close functional link between the brain systems for action, perception and semantics (Papeo et al., 2013; Caramazza et al., 2014). We find this an unconvincing argument. In fact, as already discussed in the introduction section above, important work has provided neurobiological motivation and explanation for the influence of task conditions on the presence or absence, and even on the “sign” of the interaction effects between sensorimotor and language systems. Most notably, Boulenger et al. (2006) demonstrated that action word processing can facilitate or inhibit motor movement depending on relative timing. Our present results now show that the type of motor movement can also facilitate or hinder verbal memory for action related words depending on the complexity of the motor task employed. As illustrated in Figure 3A, the inter-experimental analysis of the hand movement conditions shows a significant cross-over Word Category × Task Complexity interaction for finger movements, clearly demonstrating that changes to the complexity of the motor task reversed the pattern of interaction from interference to facilitation in the hand movement condition. The observed effect was in line with predictions generated from a neurobiological model (which we discuss in more detail below). Therefore, we believe that the documented causal effect and its task specific “sign” alteration substantially strengthen the case for causal effects of the motor system on language in particular, and for neurobiological grounded models of semantics in general.

Our present results can be seen as consistent with the idea of a “common neural basis” of motor movement and action word processing and memory. More precisely, we would like to propose that motor schemas underlying the programming of motor movements also play a role in the semantic processing and representation of action related words. Quite obviously, this does not imply that these motor representations exhaust the words’ semantic information, as typical action goals, themes and contexts play additional crucial roles. However, we would like to argue that the basic action features, that is, features of the body movement, may also play a semantic role and these features are interesting for brain language research because extremities can be mapped to specific cortical areas, thus opening fruitful perspectives for neurocognitive research (see, for example, Grisoni et al., 2016). Because the facilitation effect observed in the hand movement condition is specific to semantic word type (arm words) and since sensorimotor regions of the brain distinguish locally between body part representations (Penfield and Rasmussen, 1950; Rizzolatti and Craighero, 2004), the specific memory facilitation effect for arm-related action words by simple finger alterations can be attributed to specific sections of the primary and secondary sensorimotor cortex. The contributory facilitation effect of this system on working memory for arm-related action words suggests that motor systems are of functional relevance to the semantic processing of action words and that the relationship between action and language systems is functionally specific and sophisticated (for further discussion, see Dreyer et al., 2015). Intriguingly, recent neuroimaging confirm a role of the somatotopically organized sensorimotor cortex in semantic priming of single action words (Grisoni et al., 2016) and in semantic prediction in sentence processing (Grisoni et al., 2017).

**Figure 5:**
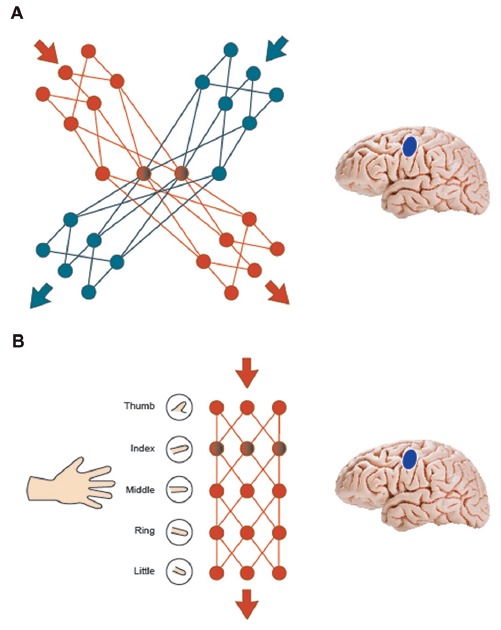
Interference and facilitation effects are illustrated using synfire chains **(A)** two similar motor sequences incompatible with each other inhibit each other by way of inhibitory connections in cortex. Complex motor movement and memory for action words compete for common processing resources in the sensorimotor cortex, resulting in local inhibition between overlapping and adjacent memory and motor circuits. **(B)** A simple motor program embedded in a more complex motor program co-activates the more complex one to a degree, resulting in priming effects. When simple finger alterations are combined with action word memory, activation from finger movements spreads to motor and memory circuits within the motor network and this co-activation facilitates memory for action words.

One may ask for an explicit neurobiological model of why a simple motor movement facilitates action word memory while a more complex movement leads to inhibition effects. The mere statement that the functional relationship between language and action systems is “flexible” (Willems and Casasanto, 2011) represents an important insight but points to the need of understanding such flexibility. There may be a mechanistic explanation for the changing of the sign as a result of varying the complexity of the motor sequence. In a neurobiological model of working memory (Fuster, 1995), lexicosemantic networks for action words include a left perisylvian component and semantic networks in motor cortex extending into arm motor cortex for arm-related words and leg motor cortex for leg-related words (Pulvermüller, 1999). Within the semantic somatotopy framework (Pulvermüller, 2001, 2005), action programs are interwoven with memory circuits for action words and such action-perception circuits become the substrate of verbal working memory for action words (Pulvermüller and Fadiga, 2010). In this model, two similar motor sequences incompatible with each other would inhibit each other by way of inhibitory connections in cortex, which are effective between locally adjacent cortical cells and neuronal assemblies (Amit and Brunel, 1997; Garagnani et al., 2008; Palm et al., 2014). Therefore, complex motor movement and memory for action words referring to actions very closely related to the movements being performed (e.g., grabbing, clapping) would compete for common processing resources in the sensorimotor cortex, resulting in local inhibition between overlapping and adjacent memory and motor circuits (Figure 5A). Note that this mechanism explains our earlier finding of inhibition between complex body-part-specific motor movements and verbal working memory for action related words related to the same body-part. Any common movements shared by two complex actions (index finger contraction in playing a melody on the piano and in grasping an apple) are typically minor compared with their substantial differences; therefore, no facilitation can be predicted. On the other hand, if a simple motor program is part of a more complex motor program (as in moving the index finger up and down and performing the same action in the context of playing the piano) then the embedded program will partly co-activate the more complex one. As the embedded program lacks components not included in the complex movement, there is less neuronal basis for inhibition and competition. Hence, when simple finger alterations are combined with processing words related to complex actions, the very simple motor program activates motor circuits for finger movement as well as other neuronal circuits in the network relevant for maintaining the memory of arm-related action words related to the motor movements. In other words, activation from finger tapping spreads to motor and memory circuits within the motor network and, similar to positive priming effects at the neurobiological level, this pre-activation facilitates memory for action words (Figure 5B). This offers an explanation for our present result of facilitation between simple finger movements and working memory processing of arm-related action words.

Forward models of motor control (e.g., Miall and Wolpert, 1996; Shadmehr et al., 2010) have been put forth to explain the relation between motor control, planning and learning. The model we advance here includes a forward or prediction component invoked in action performance and perception but goes beyond forward models of motor control by addressing the causal relationship and interaction between action and language processing (for detailed discusssion, see Pulvermüller, 2018). Our present findings and tentative accounts offer new perspectives on the explanation of well-known previous findings, which could have been seen as “contradictory,” “inconsistent” or just unexplainably “flexible.” As mentioned in the Introduction, behavioral and TMS investigations report seemingly contrasting results with regards to the sign of the interaction; some studies report inhibition effects while others report facilitation effects. In light of the current findings, it is possible that the facilitation effects observed in some studies (e.g., Pulvermüller et al., 2005; Glenberg et al., 2008b) and the interference effects reported in others (e.g., Buccino et al., 2005; Dalla Volta et al., 2009) are directly related to the nature of the motor movement involved and/or the strength of motor activation in those investigations. For instance, the facilitation of action word processing in Pulvermüller et al. (2005) was brought about by single pulse TMS to the arm and leg motor cortex. This stimulation would just slightly activate a small part of the motor cortex. Such weak activation of motor systems may not have been strong enough to interfere with action word processing, but may have primed the semantic network, resulting in the shorter reaction times observed. Similarly, the facilitation effects reported in Glenberg et al. (2008b) could be related to the simple motor movement used in that study. In the judgment task, subjects listened to sentences describing towards/away actions and had to move their index finger either towards or away from their body to press a button. As this is a basic motor movement not involving many hand/arm muscles, the faster responses to the congruent language may have arisen from the action language stimuli activating complex motor schemes of which such simple movements are part, thereby priming the subsequent motor response. As such, lingering activation from processing the action related sentences may have facilitated motor movement.

On the other hand, the interference effects reported in Dalla Volta et al. (2009) were induced by complex movements which are likely to have resulted in stronger activation of motor systems. This activation may have interfered with the complex action schemes semantically related to the arm action words. In one experiment, participants had to open their thumb and index finger by an arbitrary amount and maintain this position throughout the experiment. In another experiment by Dalla Volta et al. (2009), participants had to execute two movements in sequence that consisted of reaching for and grasping a cylinder 36 cm away then releasing the cylinder from grasp. These movements do not involve only moving the fingers, but require movement and coordination of different parts of the hand and arm, especially for grasping and releasing a cylinder placed a distance away. Such a complex motor task combined with processing action words may activate motor circuits representing incompatible complex motor schemes, which, as a result, compete with each other, thus, resulting in the inhibitory effects observed. Therefore, although the results of some of the above studies at first glance appear to be divergent, upon closer examination and on the background of the neurobiological model we advance here, they provide support for the notion of facilitatory effects between simple overt motor movement and congruent semantic language processing. Complex motor movements, on the other hand, tend to show interference with action semantic language processes.

Other models have also attempted to provide an explanation for both facilitatory and inhibitory interactions between language and action. For example, the computational model proposed by Chersi et al. (2010) shows that facilitation and interference may be “two sides of the same coin”. According to the model, motor and mirror neurons are organized in chains of neuron pools which encode short action sequences. Executing and understanding a motor sequence is a result of spreading activity within specific chains. The activation of a specific pool in a chain depends on the degree of overlap with previously activated pools of other chains, with a larger overlap resulting in a stronger influence. Therefore, pools will respond faster or slower depending on the activation phase of other pools. The authors hypothesize that neurons representing a single, elementary motor act embedded in a sequence of such acts respond even when the same act is embedded in a different sequence, a feature adopted from Abeles’ synfire chain mechanism, which gained strong support from neurophysiological studies (Abeles, 1982; Tal and Abeles, 2018). While this idea is in line with our hypotheses, computational modeling work by Chersi et al. (2010) focusses on the role of precise task timing in determining the type of functional interaction between action and language, and does not take into account motor task demands or the complexity of motor movement.

Although the issue of motor movement complexity offers some clarification on previously reported inconsistent results, there are still unexplained effects. We suggest that, within a to-be-developed elaborate model of action-language interaction effects, the factor motor complexity will be but one relevant factor, along with the relative timing of movement and language processing (Boulenger et al., 2006), the degree of attention focussing on language (Garagnani et al., 2008) and a range of other components.

## Conclusion

The present study documents facilitation effects of working memory for arm-related action words brought about by simple finger tapping and shows that the direction of the effect of motor movement on action word memory can be reversed from inhibition to facilitation by changing the complexity of the motor task. By manipulating the sign of the interaction effect, this finding demonstrates the important functional specificity and flexibility of motor systems in action word memory and adds to a growing body of literature demonstrating functional links between the neural bases of action and language.

## Author Contributions

Both ZS and FP contributed to the design of the experiment and contributed to the manuscript. ZS collected the data and performed the analyses.

## Conflict of Interest Statement

The authors declare that the research was conducted in the absence of any commercial or financial relationships that could be construed as a potential conflict of interest.
